# Engagement with a Web-Based Health Promotion Intervention among Vocational School Students: A Secondary User and Usage Analysis

**DOI:** 10.3390/ijerph17072180

**Published:** 2020-03-25

**Authors:** Gerrit Stassen, Christopher Grieben, Ingo Froböse, Andrea Schaller

**Affiliations:** 1 Working Group Physical Activity-Related Prevention Research, Institute of Movement Therapy and Movement-oriented Prevention and Rehabilitation, German Sport University Cologne, D-50933 Cologne, Germany; a.schaller@dshs-koeln.de; 2 Department 1: Movement-oriented Prevention and Rehabilitation Sciences, Institute of Movement Therapy and Movement-oriented Prevention and Rehabilitation, German Sport University Cologne, D-50933 Cologne, Germany; c.grieben@dshs-koeln.de (C.G.); froboese@dshs-koeln.de (I.F.)

**Keywords:** web-based platform, vocational school students, initial face-to-face contact, engagement, logistic regression, log-data visualization

## Abstract

Engagement with web-based interventions is both generally low and typically declining. Visits and revisits remain a challenge. Based on log data of a web-based cluster randomized controlled trial conducted in vocational schools, the present secondary analysis aimed to identify influencing factors on initially logging in to a health promotion platform among young adults and to examine the engagement over the course of an eight-week intervention. Data of 336 students (62.2% female, age span 18–25) from two intervention arms (web-based intervention and web-based intervention with an additional initial face-to-face contact) was included. Binary logistic regression and log-data visualization were performed. An additional initial face-to-face contact (odds ratio (OR) = 2.971, *p* = 0.005), female sex (OR = 2.237, *p* = 0.046) and the health-related skill “dealing with health information” (OR = 2.179, *p* = 0.030) significantly increased the likelihood of initially logging in. Other variables showed no influence. 16.6% of all potential users logged in at least once, of which 57.4% revisited the platform. Most logins were tracked at the beginning of the intervention and repeated engagement was low. To increase the engagement with web-based interventions, health-related skills should be fostered. In addition, a strategy could be to interlink comparable interventions in vocational schools more regularly with everyday teaching through multi-component interventions.

## 1. Introduction

As today’s leading medium, the internet offers great potential and wide reach for health promotion [1,2]. Especially among younger target groups, it is already established as a primary source of health information [3,4,5].

Nevertheless, engagement with web-based interventions for health promotion is generally low and high attrition rates are typical [6,7,8]. At the beginning of an intervention, three key stages are usually important: that users access the website (or a comparable web-based program), stay there, and revisit it [9].

The combination of digital and analogue study components, e.g., through an initial kick-off or mixed intervention designs, can increase engagement with web-based interventions [10,11]. Other intervention components that have been shown to have a positive impact on engagement include tailored prompts or reminders and regular updates [12,13,14,15].

However, it can be observed that actual users are often not necessarily those who should be using a web-based health program [16,17], which reconfirms the inverse care law [18]. A higher level of engagement with in web-based health programs is again associated with, e.g.,: female sex, normal weight, compliance with guidelines for healthy behavior, and higher health literacy [17,19,20,21,22].

A more difficult target group to reach is young adults, especially young men, who use the internet more often in general and more often as a source of health information, but who often show low levels of use and revisits to web-based interventions [7,17,21,23]. In addition, young adults are an often-overlooked target group in health promotion [24,25], although they have high societal relevance as a future workforce [26,27] in increasingly aging Western societies [28]. At the same time, the phase of “emerging adulthood” (age span 18–25 years) is essential for personality development and the exploration of possibilities [29] and has a major impact on the manifestation of healthy behaviors and well-being [30,31,32,33]. A promising strategy for addressing young target groups through web-based interventions is integration into social contexts, e.g., in educational institutions [13].

Against the background of the great potential of web-based health promotion, but also low and decreasing participation, user and usage analyses for the identification of (non) user characteristics as well as engagement patterns are of increasing importance [34,35,36].

Based on a web-based intervention cluster randomized controlled trial conducted in vocational schools [37,38], the following research questions were investigated: (1) What are influencing factors among vocational school students on initially logging in to a web-based health promotion platform? (2) What is the overall engagement and the engagement like during the course of an intervention in a web-based intervention group compared to a group with an additional initial face-to-face contact?

## 2. Materials and Methods

### 2.1. Study Design and Participants

**Figure 1:**
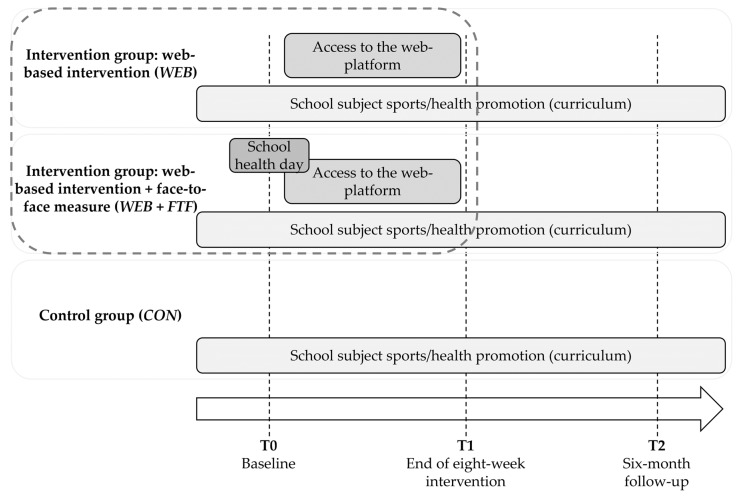
Study design (cluster randomized controlled trial, encircled: study segments of the secondary analysis).

The present study involved secondary analyses of the main phase of the WebApp study [37], which was conducted as a three-armed cluster randomized controlled trial with three measuring points (T0—baseline (start of the intervention), T1—end of eight-week intervention, T2—six-month follow-up) starting in February/March 2017 (Figure 1). WebApp dealt with the health literacy promotion of vocational school students. Three forms (33 classes in total) from three different schools from three different urban districts in Cologne, North Rhine-Westphalia, Germany, were recruited based on project-related cooperation agreements with the German Sports University Cologne and randomly assigned to the following study conditions: web-based intervention (*WEB*), web-based intervention with an additional initial face-to-face contact (*WEB + FTF*), or control (*CON*, no access to the web-based platform). All participants were completing commercial vocational training (typically lasting 3 years) and were in their first year of training, except for one class.

During the study, all classes continued to participate in regular lessons of the school subject sports/health promotion which—according to the curriculum of the federal state of North Rhine-Westphalia [39]—is compulsory across all training programs and aims to contribute to personality development and to support a self-determined health-promoting way of living. Participation in the study was voluntary and did not interfere with regular schooling for the duration of the study.

In the evaluation of the WebApp main phase [38], neither WEB nor WEB + FTF was more effective in terms of health literacy promotion compared with CON. None of the study conditions showed a significant improvement in health literacy.

**Figure 2:**
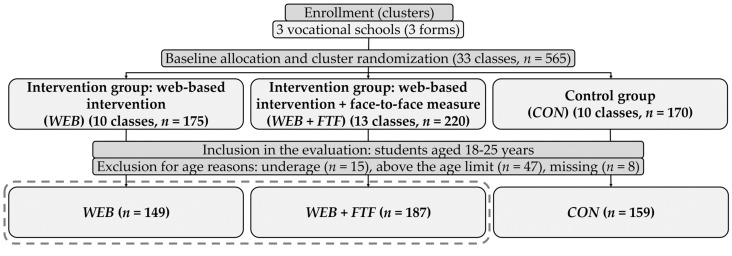
Analysis inclusion flow chart (encircled: study conditions of the secondary analysis).

As in the evaluation of the study’s main phase [38], the present user and usage analyses included only students aged 18–25 years following Arnett’s conception of “emerging adulthood” [29]. Data of underaged and students outside the age span was not included. The corresponding baseline sample (33 classes, *n* = 565, *n* = 495 in the age span 18–25 years) was reduced to the two intervention groups and the intervention period (Figure 1), resulting in a final sample of *n* = 336 (23 classes, *WEB*: 149, *WEB + FTF*: 187) (Figure 2). Written informed consent was obtained from all participants in the sample. The Ethics Committee of the German Sport University Cologne has approved the study (reference: 118/2015).

### 2.2. Intervention Groups

#### 2.2.1. Web-Based Intervention (WEB)

The main objective of the web-based intervention was to strengthen competencies regarding a healthy lifestyle. For the realization of the web-based platform, an e-learning software (Talentsoft GmbH, Cologne, Germany) was used. The software provided responsive design. The web-based platform was developed based on focus groups with vocational school students, which were conducted in an earlier project stage (results presented elsewhere [40]).

**Figure 3:**
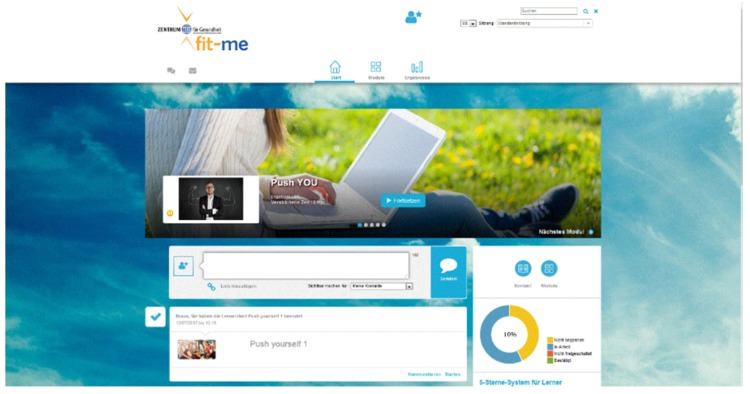
Homepage of the web-based platform.

Interactive functions were integrated (profile function, private messages, newsfeed). On the homepage the timeline, updates, and personal progress were displayed and different sections of the platform were accessible via the header, including the learning modules (Figure 3).

The content section included modules covering seven specific topics: general information (main focus: physical activity), clarification of misinformation, healthy nutrition, quick recipes, motivation, check-ups, and quizzes. The content section was updated about once a week, seven times during the eight-week intervention. The content was designed to be interactive, using various multimedia formats and was tailored to the target group in terms of language, scope and complexity.

Structure and content were identical for both groups, but the two platforms were independent of each other. After the baseline measurements, the participants of both intervention groups received invitation emails with individual accounts. During the intervention, all users received short email reminders describing the updated contents about once a week.

#### 2.2.2. Web-Based Intervention with an Initial Face-to-Face Contact (*WEB + FTF*)

In addition to platform access (*WEB*), a school health day was conducted during a regular school day (obligatory participation) before the start of the intervention (*WEB + FTF*). The topics were occupational health management, brief relaxation at the workplace and stress management, healthy nutrition, fitness tests, health check-ups, and the presentation of the platform. The aim was to sensitize the students to health topics and to present the platform through personal contact.

### 2.3. Log-Data

Log-data was automatically tracked by the e-learning software and could be extracted by the authors via an admin function. The software tracked the user ID and the login time. Every listed login during the intervention period was included in the analyses.

### 2.4. Measures

All measurements (paper-pencil questionnaires) took place during regular school lessons or the school health day, respectively, and were voluntary.

Sex, height and weight for body mass index (BMI) calculation were based upon self-report.

For measuring work ability, the German short version of the Work Ability Index was used (WAI) [41]. The WAI results in a score from 7–49 which—following the (re)classifications of Kujala and colleagues for young employees [42]—allows a classification into four categories: poor work ability (score 7–36), moderate (37–40), good (41–44), excellent (45–49). The questionnaire relates well with objective measurements [43,44], has an acceptable test-retest reliability [45], and prognostic value regarding sickness absence [46].

Subjective psychological well-being was assessed with the German version of the 5-item World Health Organization Well-Being Index (WHO-5) [47]. The sum score ranges from 0–25 and is multiplied by 4. A score of ≤ 50 indicates a reduced well-being [48]. The questionnaire has been shown to have a high clinimetric validity and is established as tool for comparing well-being between groups [48,49].

Physical activity was measured using a German translation of the one single question by Milton and colleagues (“In the past week, on how many days have you done a total of 30 minutes or more of physical activity, which was enough to raise your breathing rate.”) [50]. This single-item assessment tool has a strong test-retest reliability (r = 0.86) and modest validity (r = 0.53) against the Global Physical Activity Questionnaire in an adult population (18–64 years) [50]. It enables assessment of whether respondents meet the recommendations of at least five days of 30 minutes of physical activity per week [51].

Health literacy was measured using Lenartz’s German questionnaire on health literacy. The questionnaire depicts the structural model of health literacy developed by Lenartz, Soellner and colleagues [52,53] and comprises 29 items representing the six “advanced skills” of the model: “self-perception”, “proactive approach to health”, “dealing with health information”, “self-control”, “self-regulation”, and “communication and cooperation”. The possible answers are “not correct at all”, “rather not correct”, “rather correct”, and “correct” (scale 1–4). The values are calculated by averaging [52]. The questionnaire proved to be reliable (Cronbach’s α for the six “advanced skills” = 0.70–0.89) [53] and the model was cross-validated with different samples [52,53,54].

### 2.5. Statistical Analyses and Visualizations

A binary logistic regression was used to answer research question (1). Initially logging in to the web-based platform was used as the dependent variable (at least one login vs. no login). Intervention group (*WEB + FTF* vs. *WEB*), sex (female vs. male), BMI (<25 normal weight vs. ≥25 overweight), well-being (score > 50 vs. ≤ 50), work ability (excellent/good vs. moderate/poor), physical activity (≥5 days of 30 minutes per week vs. <5 days), and the six “advanced skills” of the structural model of health literacy were included as independent variables. The dichotomizations were based on established cut-off values (see 2.4. Measures) to analyze the influence of various risk factors. Cases with missing values were excluded from the analysis.

Independent samples t-tests or Mann–Whitney U-tests, respectively, and chi-square were used to examine research question (2) regarding the proportion of users, the proportion of revisitors, and the login frequency. Furthermore, for each time point of the web-based intervention, i.e., for the time after the invitation e-mail and after subsequent updates, the relative proportion of users within the two study conditions (students with at least one login divided by the size of the respective intervention group) was calculated. A scatter diagram was created for both intervention groups, with the users of both interventions groups on the y-axis and the time points of the web-based intervention on the x-axis, to visualize at which time points each user logged in at least once, with the lines in between indicating repeated engagement over multiple time points.

The significance level was set at *p* < 0.05 for all tests. The data was analyzed using IBM SPSS Statistics 26 (IBM Corp., Armonk, NY, USA).

## 3. Results

Of the sample (Table 1), the majority (62.2%) were female and mean age was 20.6 ± 1.9 years. 56.0% had a reduced well-being, 52.2% showed “poor”/“moderate” work ability, and 71.3% did not meet the physical activity recommendations. Scores of the six “advanced skills” of the structural model of health literacy varied between 2.6–3.0.

### 3.1. Influencing Factors on Intially Logging in

The logistic regression showed that the model as a whole (χ² (12) = 21.625, *p* = 0.042) was significant (Table 2). Intervention group and female sex increased the likelihood of initially logging in by 2.971 and 2.237 times, respectively, and if “dealing with health information” increased by one unit, the likelihood of initially logging in increased 2.179 times. Other variables showed no influence.

### 3.2. Engagement

Of all potential users, 16.1% (54/336) logged in at least once, and 57.4% of those revisited the platform. In *WEB*, 9.4% (14/149) logged in at least once, whereas in *WEB + FTF* 21.4 % (40/187) logged in, which means a significantly higher proportion of users (χ² (1) = 8.845, *p* = 0.003) (Table 3). In terms of proportion of revisitors, the groups did not differ. The login frequency did not differ, despite a tendency towards *WEB*. In total, 146 logins were tracked (*WEB*: 45, *WEB + FTF*: 101).

**Figure 4:**
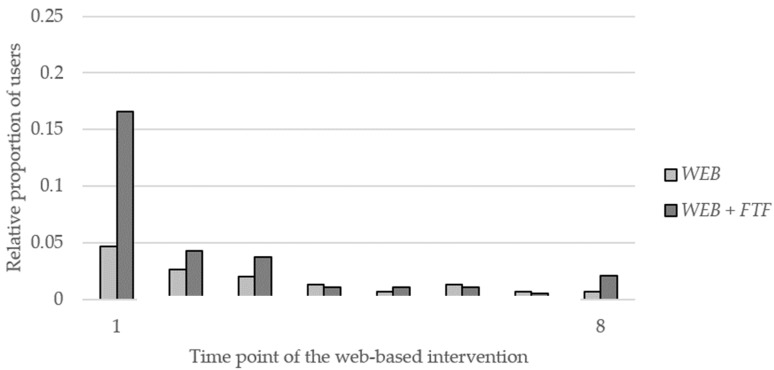
Relative proportion of users during the web-based intervention (time point: 1—after invitation emails; 2–8—after subsequent update).

Figure 4 shows the course of participation based on the relative proportion of users (students with at least one login divided by the size of the respective intervention group). The highest participation was from *WEB + FTF* after the invitation emails (16.6%). Apart from this, the relative proportion of users within the two intervention groups was always below 5% for each subsequent time point of the web-based intervention, i.e., after the subsequent updates. In both groups, the majority of the total number of logins within the group were tracked after the invitation email and before the first update (*WEB*: 35.6%, *WEB + FTF*: 59.4%).

**Figure 5:**
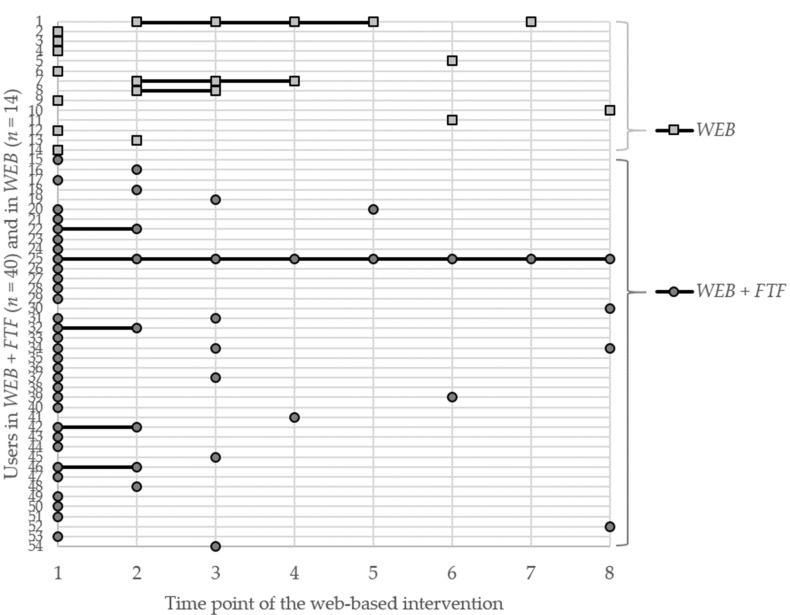
Scatter diagram visualizing the time points of the web-based intervention at which each user logged in at least once (time point: 1—after invitation emails; 2–8—after subsequent update).

The scatter diagram (Figure 5) visualizes at which time points of the web-based intervention each user logged in at least once (squares: *WEB*, dots: *WEB+FTF*, lines in between indicating repeated engagement). For eight users (14.8%), repeated engagement could be observed between at least two time points of the web-based intervention. One user in *WEB+FTF* logged in after both the invitation email and all seven updates.

## 4. Discussion

Engagement with the web-based intervention was low, both in terms of the proportion of users and revisitors and in the further course of the study. An additional initial face-to-face contact, female sex, and a higher score in “dealing with health information” significantly increased the likelihood of initially logging in to the web-based intervention platform.

With regard to the baseline values for overweight [56], work ability [42], well-being [57], meeting physical activity recommendations [58], and health literacy [52], it can be stated that they are comparable to German surveys or corresponding studies with young adults, which makes the study population to a certain extent representative of the target group of vocational school students. The higher number of female participants is influenced by commercial vocational training, which is more popular among women [59]. The generalizability of the results is limited with regard to other age groups or settings. However, since a majority of web-based health promotion studies targeting young adults are conducted in university settings [25,60,61], the present study has a great added value with regard to non-academic settings.

In web-based health promotion in general, it has already been proven that potential male users are more difficult to reach and show higher dropout rates and less use of online health information [7,21,23,62]. Moreover, young men are considered a partially neglected target group in health promotion in general [63,64,65]. Specific surveys of this subgroup show that a combination of personal and web-based appointments could be promising [66].

Regarding the structural model of health literacy, only “dealing with health information”—the ability to understand and integrate health-related information into one’s personal life—had an impact on initially logging in to the web-based platform. Actively managing one’s health is associated with use of the internet for information about healthcare and healthy lifestyle information [67]. In addition, low health literacy is negatively associated with the evaluation of online health information [68]. On the other hand, due to the non-impact of the other “advanced skills” of the structural model, this result should be viewed with caution, as students with a higher levels of health literacy may not have seen the need to use a (further) source of information. The use of online resources for health information specifically requires eHealth literacy skills, which should be trained in young adults [69]. Further research is needed to explore in detail the relationship between health literacy and the use of target group-oriented web-based health information.

The fact that increased BMI, reduced well-being, reduced work ability, and insufficient physical activity and all other skills of the health literacy model did not influence initially logging may underline that those who use a (digital) health promotion measure are not always the people who should be doing so. Therefore, it remains challenging to reach risk subgroups within heterogeneous target groups, which is why special attention should be paid to less responsive groups, both in the planning and implementation of studies [21,70].

Regarding the implementation, the analyses show that an additional initial face-to-face contact can positively influence the absolute proportion of users who log on to a web-based platform. However, in both intervention groups the majority of logins were tracked in the time between the invitation emails and the first update; in *WEB + FTF* even more than half, so that no further sustainable effect can be deduced in the present study with regard to engagement. The purposeful and also repetitive and continued integration of face-to-face components at multiple time points of a web-based intervention, e.g., as a blended intervention, can lead to an increase in adherence [14]. Blending online and offline learning content within a curriculum is considered more valuable than online learning alone [71]. Studies show that in educational institutions the possibility of using a web-based health program during teaching time and not only during leisure time is a crucial factor for regular usage [72,73]. The integration of web-based measures into everyday school life can reduce barriers to use, e.g., low motivation [74].

Accordingly, it must be considered that at least one further face-to-face meeting, e.g., in the middle of the study, might have been beneficial. As stand-alone web-based interventions appear to be less effective than multi-component interventions [75], there may be increased potential when combined with more regular face-to-face contact.

In a larger context, however, research has shown that web-based measures without initial face-to-face contact have a comparable effectiveness to those without, especially when the initial appointment is usually only a technical instruction [76]. This has important implications for the dissemination of interventions, since although participation following face-to-face contacts may be higher in comparison, the overall reach increases significantly without [76]. Accordingly, digital and face-to-face measures should only be combined if the combination has added value in terms of the intervention objective.

Since in both groups the majority of logins and the highest relative proportion of users were tracked in the first week of the intervention, following which usage decreased, it becomes clear that reminders and updates could not attenuate attrition in the present study. The low number of users for whom repeated engagement could be observed confirms this. Research suggests that periodic reminders and tailored prompts to use (web-based) health interventions can have a positive impact on engagement [77,78,79], especially if they are additionally combined with personal contact [80]; neither of these components was used in the present study. The optimal frequency still needs to be investigated [77,81], because a high frequency can also lead to “fatigue” and thus to ignoring [7]. Studies also show that website updates have positive effects on the proportion of revisitors [12,14,65], but probably not if the first visit to a platform was not engaging.

Furthermore, different interests and needs of specific subgroups should be considered and specifically addressed. Otherwise, the intervention approach might not be appropriate for any group in retrospect, since a “one-size-fits-all” approach is unlikely to satisfy the preferences of the various users [82]. Future interventions for young user groups should be app-oriented due to current usage behavior and should include personalized and tailored feedback and content, self-monitoring and goal-setting, and social interaction and comparison [82,83,84]. Similar components were also mentioned in the focus groups in an earlier project phase of the WebApp study [40], but could not be fully realized in the main phase of the study due to technical reasons and lack of IT resources. Participation processes can quickly accumulate broad ideas, but these may no longer be within the scope of what is feasible. A clear, reduced framework and appropriate objectives should therefore already be set before an intervention is developed.

When planning web-based measures for young adults, the special phase of life of this target group should always be carefully considered. Young adults, including vocational school students, are in a life phase characterized by exploration and self-orientation [29], especially at the beginning of a training course or study. Factors such as pleasure, appearance and well-being are much more important for this very specific target group than prevention and health promotion [85,86]. To prevent inappropriate addressing and wrong framing of health information [85,86,87], very target group-specific web-based strategies are necessary.

Despite previous target group participation, the final platform might have been too information-heavy and not low-threshold enough. Even though the content was designed to be interactive and tailored to the target group, the platform approach was most likely unsuitable for the current usage and information behavior of the target group. Potential users must be able to recognize a personal benefit and, in the best case, be well entertained to some extent in order to engage with prevention and health.

All in all, the low user rates underline the challenge of target group-oriented web-based prevention measures for young adults. The low login frequency does not indicate stable use, and, looking at the course of the study, users lost interest after a short time. The generally well-known phenomenon of high dropout rates in web-based interventions was confirmed. In future interventions, attention should be paid to the crucial moments in terms of engagement, namely the hurdle of the first visit, the challenge of ensuring that users stay with an offer and that they return [9]. Interdisciplinarity is more important than ever to design modern digital interventions and to keep pace with technological and societal developments [88,89,90]. Nonetheless, insights into measures with a rather low engagement levels continue to be very valuable in driving forward the development of future measures [8,91].

### Limitations

First, the selected usage parameter, i.e., logins, gives a broad insight into usage frequency but not into deeper engagement [92,93]. Usage or engagement can be distinguished between extent of usage as behavior, i.e., amount or frequency, and subjective experience, e.g., attention and interest [35]. The scientific discussion on the operationalization of engagement and the selection of use metrics is ongoing [35,94,95]. Since we have taken logins as a meaningful measure for our secondary post-hoc usage analysis [96] to get a general overview of the participation, we cannot draw any conclusions about individual experience. Against the background of the known high drop-out rates, in-depth analyses of the perception and usability of web-based measures for potential users are necessary. The Technology Acceptance Model [97] or the System Usability Scale [98] offer possible approaches. Also, no intended engagement use was predefined [98,99], as the intervention was not designed with a strict modular structure, e.g., in the sense of a teaching series.

Secondly, regarding the factors that influence initially logging in, the regression analysis did not integrate further variables that are known to influencing engagement, such as intention and motivation for behavior change [34,79,100], perceiving personal relevance [101], or social class and education level [102]. Future studies should consider these aspects in young adults. In addition, both the WAI and the WHO-5 are established instruments, but the use of the one single question to measure physical activity can in turn be viewed critically. Only a few instruments for measuring physical activity show sufficient reliability and validity [103,104]. Furthermore, the selected and internationally barely established health competence questionnaire makes comparisons with other studies difficult. The questionnaire remains to be translated and further validated. Nevertheless, it offers an insight into individual skills within a health literacy model and starting points for future studies. The operationalization of health literacy and the choice of a measurement instrument remains a subject of current discussions [105,106].

Thirdly, engagement over the course of the study was only examined descriptively. Due to the open intervention format, which means that the updates did not build on each other, further statistical analyses of the course of logins were not carried out. Instead, log-data visualization was used to get an impression of the course as an alternative way of investigating engagement [107,108]. In strictly modular or sequenced interventions, survival analysis methods, e.g., Kaplan–Meier analysis, allow for adherence and attrition analyses [8].

## 5. Conclusions

Young adults are a heterogeneous target group and find themselves in a special phase of life, which is why a “one-size-fits-all” intervention approach does not seem very promising. Therefore, especially risk subgroups, such as young men, should be addressed more specifically.

Since prevention and health promotion play a subordinate role among vocational school students and young adults in general, a pronounced interest in health topics or a high level of intention to use (web-based) health promotion measures cannot necessarily be assumed. To increase the engagement with web-based interventions, however, health-related skills, such as the ability to understand and integrate health-related information into one’s personal life, should be fostered.

With regard to implementation, it can be stated that an initial face-to-face contact can have a positive influence on the absolute proportion of users, but does not necessarily lead to a long-term stable engagement, even if reminders and updates are used. A possible strategy could be to interlink future web-based interventions in vocational schools more regularly with everyday teaching by means of multi-component interventions, so that the advantages of both sides (more frequent face-to-face contact and a contemporary approach) can complement each other.

## Figures and Tables

**Table 1:** Baseline values (*n* = 336).

Characteristic, Outcome	*n* (%) ^1^/Mean ± SD
Sex (female)	209 (62.2)
Age (years)	20.6 ± 1.9
BMI	23.8 ± 4.7
Overweight (BMI ≥ 25) ^2^	101 (30.7)
WAI score (range 7–49)	39.6 ± 4.6
Poor/moderate work ability (score ≤ 40) ^3^	168 (52.2)
WHO-5 score (range 0–100)	47.5 ± 17.5
Reduced well-being (score ≤ 50) ^4^	187 (56.0)
Days/week ≥ 30 minutes of physical activity	3.2 ± 2.0
Not meeting recommendations (<5 days/week) ^5^	239 (71.3)
Structural model of health literacy: “advanced skills” (scale 1–4)	
Self-perception	3.0 ± 0.4
Proactive approach to health	2.6 ± 0.6
Dealing with health information	2.8 ± 0.6
Self-control	2.9 ± 0.5
Self-regulation	2.6 ± 0.6
Communication and cooperation	2.6 ± 0.6

Note: ^1^ Valid percentages due to missing data. SD—standard deviation. BMI—body mass index. WAI—Work Ability Index. WHO-5—World Health Organization Well-Being Index. ^2^ [55]. ^3^ [42]. ^4^ [48]. ^5^ [51].

**Table 2:** Logistic regression model with initially logging in ^1^ as dependent variable (*n* = 289).

Factor	B	SE	*p*	OR	95%-CI
Intervention group (*WEB + FTF* vs. *WEB*)	1.089	0.384	0.005 **	2.971	1.399; 6.309
Sex (female vs. male)	0.805	0.404	0.046 *	2.237	1.014; 4.934
BMI (<25 vs. ≥25)	−0.254	0.387	0.512	0.776	0.398; 1.788
Work Ability (WAI score > 40 vs. ≤40)	−0.170	0.383	0.657	0.844	0.398; 1.788
Well-being (WHO-5 score > 50 vs. ≤50)	0.120	0.404	0.767	1.127	0.511; 2.487
Physical activity (≥5 days vs. <5)	−0.215	0.403	0.593	0.806	0.366; 1.775
Self-perception	0.056	0.431	0.896	1.058	0.455; 2.462
Proactive approach to health	−0.017	0.318	0.957	0.983	0.527; 1.833
Dealing with health information	0.799	0.358	0.030 *	2.179	1.081; 4.393
Self-control	0.079	0.390	0.840	1.082	0.504; 2.324
Self-regulation	−0.213	0.339	0.530	0.808	0.415; 1.572
Communication and cooperation	0.071	0.306	0.816	1.074	0.589; 1.958

Note: ^1^ Dependent variable: at least one login vs. no login. B—unstandardized regression coefficient, SE—standard error, OR—odds ratio, CI—confidence interval. BMI—body mass index. WAI—Work Ability Index. WHO-5—World Health Organization Well-Being Index. *p* < 0.05 *. *p* < 0.01 **. Nagelkerke’s *R^2^* = 0.123.

**Table 3:** Overall use of the web-based platform.

Measure	*WEB* n(%) ^1^/Mean ± SD	*WEB + FTF* n (%) ^1^/Mean ± SD	*p*
Proportion of users ^2^	14 (9.4)	40 (21.4)	0.003 **
Proportion of revisitors ^3^	9 (64.3)	22 (55.0)	0.545
Login frequency ^3^	3.2 ± 2.3	2.5 ± 4.1	0.124

Note: ^1^ Valid percentages due to missing data. SD—standard deviation. ^2^ At least one login/initial login rate. ^3^ At least two logins. ^3^ Among proportion of users. *p* < 0.01 **.
